# Supplementary material for: Neutrophil stunning by metoprolol reduces infarct size
Source: Nat Commun. 2017 Apr 18;8:14780. doi: 10.1038/ncomms14780 (PMC5399300; doi:10.1038/ncomms14780)
Supplement: Supplementary Information — Supplementary Figures and Supplementary Table. [file ncomms14780-s1.pdf]

Supplementary Figure 1. Metoprolol administration during ongoing AMI reduces MVO in STEMI patients

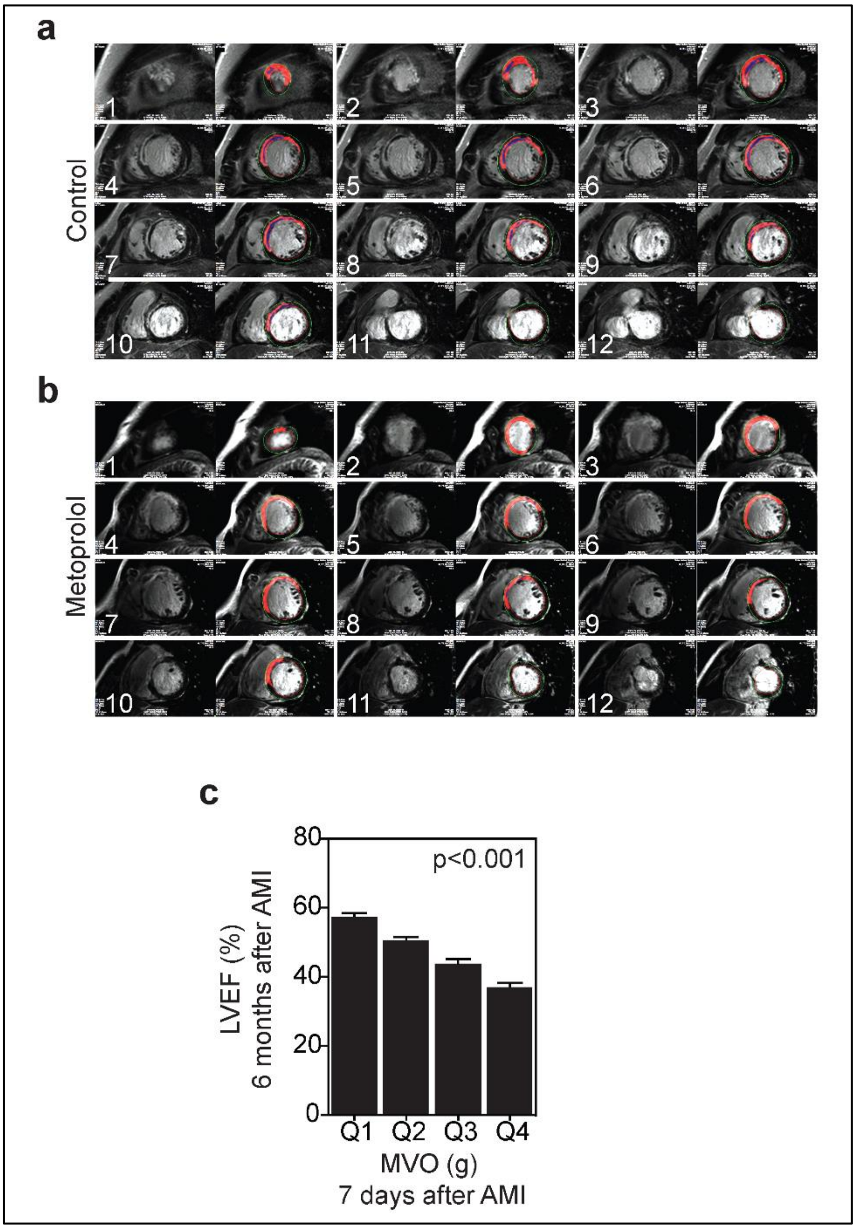

(a, b) Complete representative CMR exams (short-axis covering the entire left ventricle (LV) from base to apex), showing significant differences in one-week post-AMI MVO evaluated in a control patient (upper panel) and a metoprolol-treated patient (lower panel). MVO was defined as the absence of contrast wash-in inside the delayed gadolinium-enhanced area (red, automatic quantification). **(c)** Long-term cardiac function (left ventricular ejection fraction, LVEF) evaluated by CMR 6 months after AMI (n=202) according to quartiles of MVO extent evaluated as in panel a. LVEF was significantly smaller in patients with larger extent of MVO. P value for linear trend is shown. Data are means ± s.e.m.

Supplementary Figure 2. Metoprolol reduces neutrophil infiltration in injured hearts

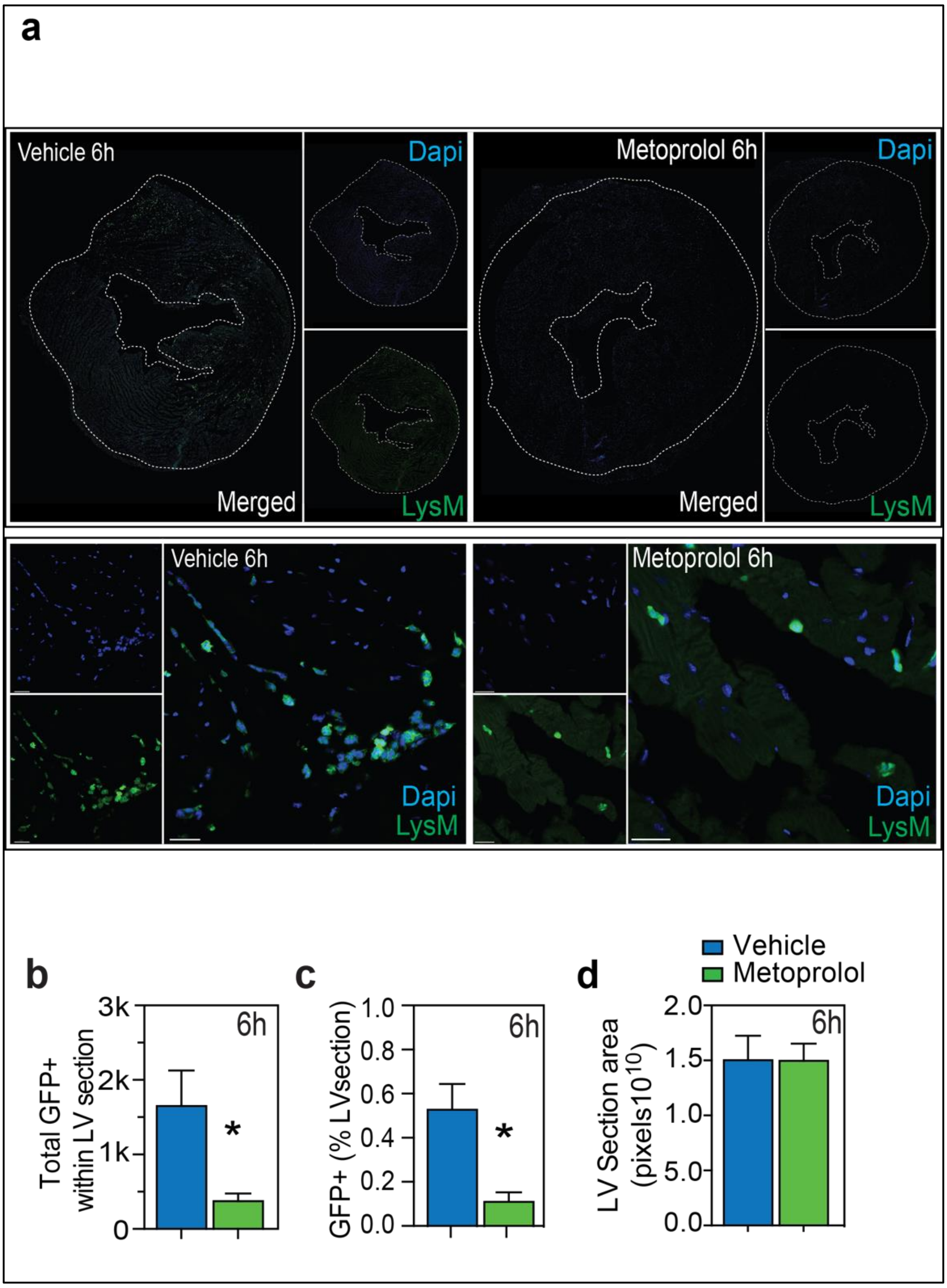

(a) Representative confocal microscopy images of complete left ventricle (LV) sections at 6h after reperfusion. Myeloid infiltration (LysM+, green) is massive within the injured myocardium of hearts from vehicle-treated mice as compared to those from metoprolol-treated mice. Lower panels represent a magnification illustrating accumulation of myeloid cells in a vessel. **(b)** Average total positive LysM+ pixels within the complete LV section. **(c)** Average LysM+ as percentage of LV section area. **(d)** Mean complete LV sections area. Data are means ± s.e.m. * $p<0.05$. Comparison was determined by the nonparametric Wilcoxon-Mann–Whitney test.

Supplementary Figure 3. Monocyte infiltrative dynamics in the myocardium

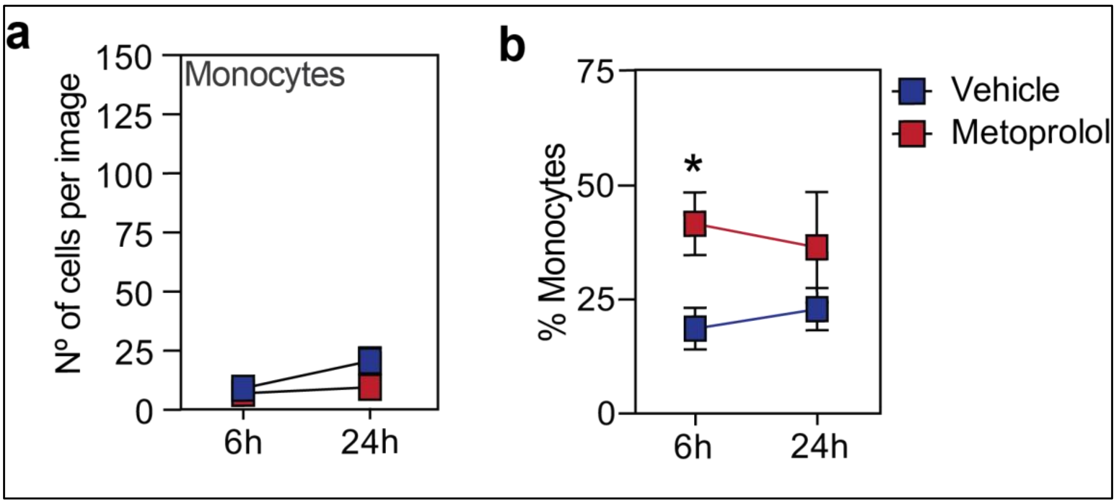

(a) Monocyte infiltration dynamics into the injured myocardium within the first 24h of reperfusion; n=5 animals per group. **(b)** Percentage of monocytes within the myeloid–derived population.

Supplementary Figure 4. Neutrophils express β1AR

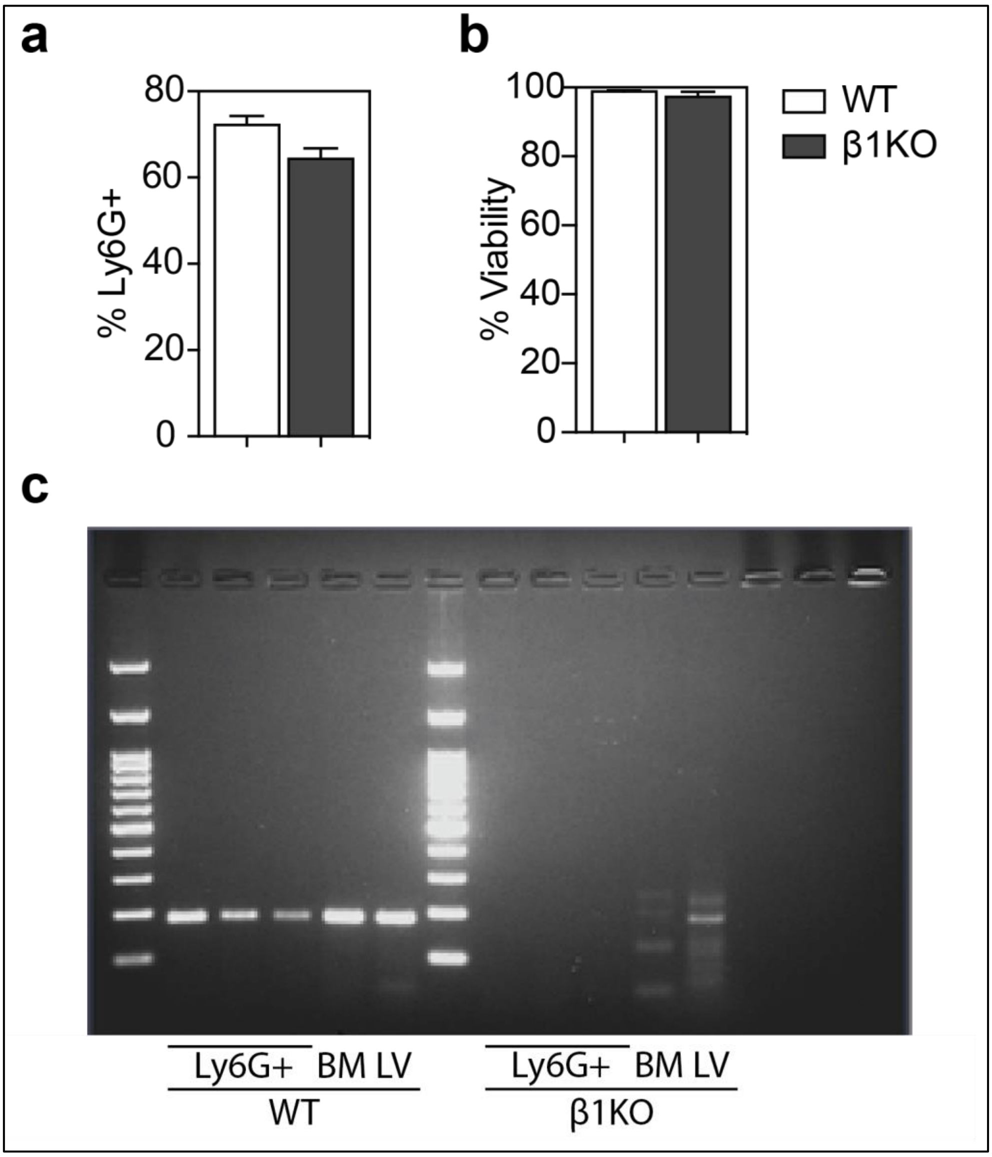

(a, b) Purity and viability of mouse blood neutrophils (LY6G+) evaluated by flow cytometry. **(c)** Agarose gel electrophoresis of PCR products, showing expression of *ADRB1* in mouse left ventricle (LV), bone marrow (BM), and blood neutrophils (Ly6G+). *ADRB1*-knockout (β1KO) mice were used as a negative control.

Supplementary Figure 5. Neutrophil migration inhibition is independent to *ADRB2*

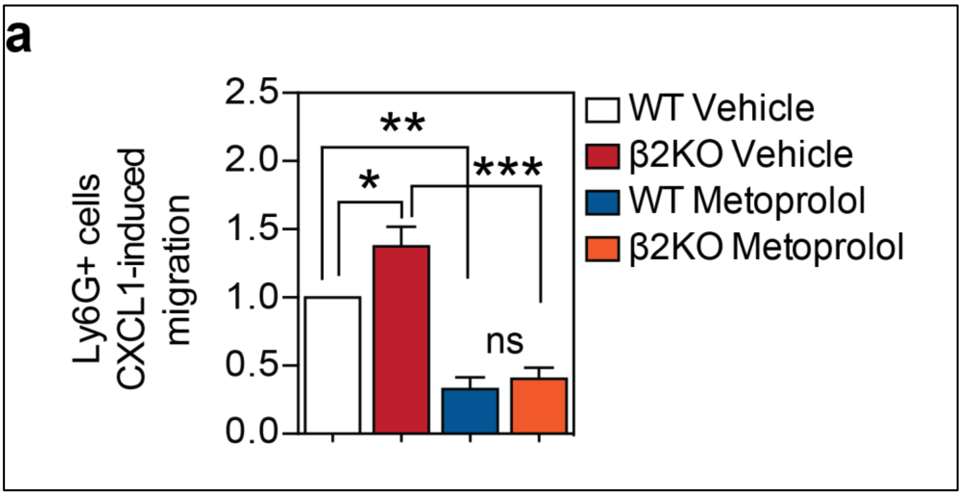

Effect of metoprolol on CXCL1-induced migration of fresh isolated primary neutrophils (Ly6G+) from *ADRB2*-knockout (β2KO) mice. CXCL1-stimulated cells were incubated with vehicle or metoprolol (10μM), n=5 independent experiments; Data are means ± s.e.m. * $p<0.05$; ** $p<0.01$, Comparisons were performed using the one-way ANOVA and Holm Sidak's post-hoc multiple comparisons method.

Supplementary Figure 6. Metoprolol blocks neutrophil infiltration through *ADRB1* blockade

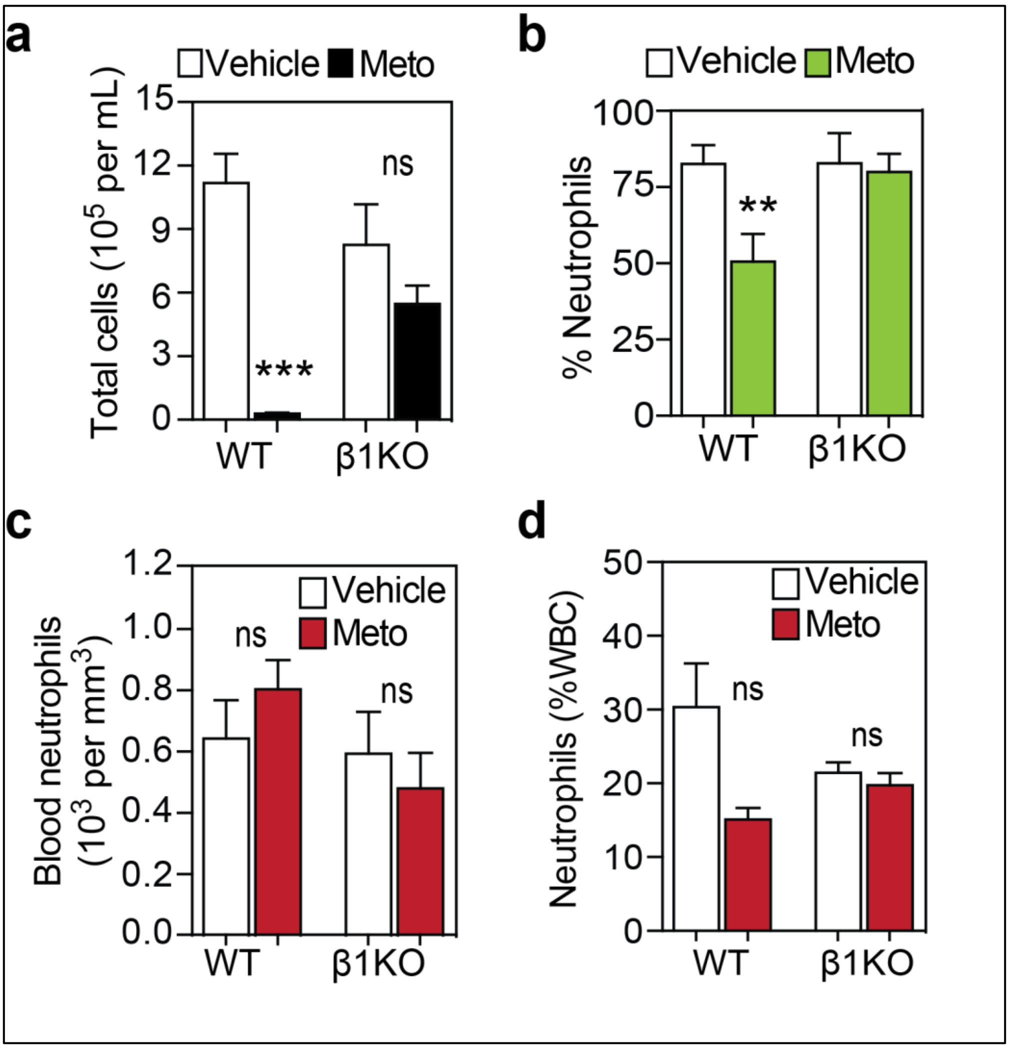

(a) Absolute leucocyte number per mL of infiltrate 16h after intraperitoneal thioglycolate injection in WT mice (n= 7-9) or *ADRB1*-knockout (β1KO) mice (n=5) randomized to receive either IV metoprolol or vehicle. **(b)** Neutrophils (CD115neg; GR1+) as a percentage of the total cells evaluated. **(c)** Peripheral blood neutrophil count. **(d)** Neutrophils as a percentage of white blood cells evaluated. Data are means ± s.e.m. ** $p<0.01$; *** $p<0.001$, determined by the nonparametric Wilcoxon-Mann–Whitney test for each panel; ns, non-significant.

Supplementary Figure 7. Flow cytometry assessment of bone marrow transplant engraftment.

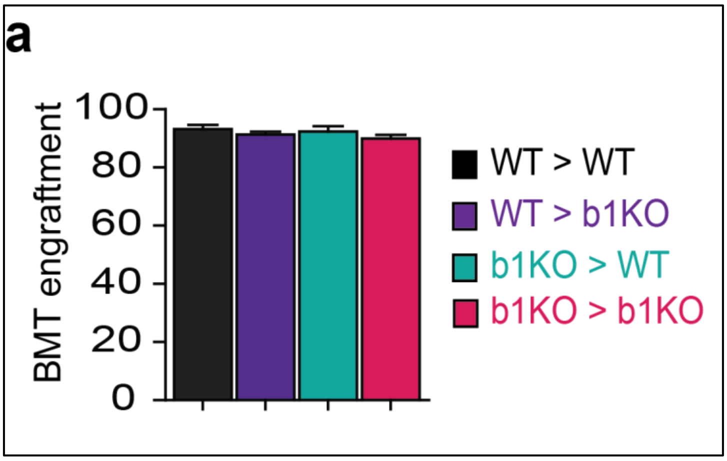

Flow cytometry assessment of representative bone marrow transplant engraftment between 4 chimeric groups evaluated. b1KO, stands for *ADRB1*- knockout. n=10. Data are means ± s.e.m.

Supplementary Figure 8. Metoprolol effect on human platelet function

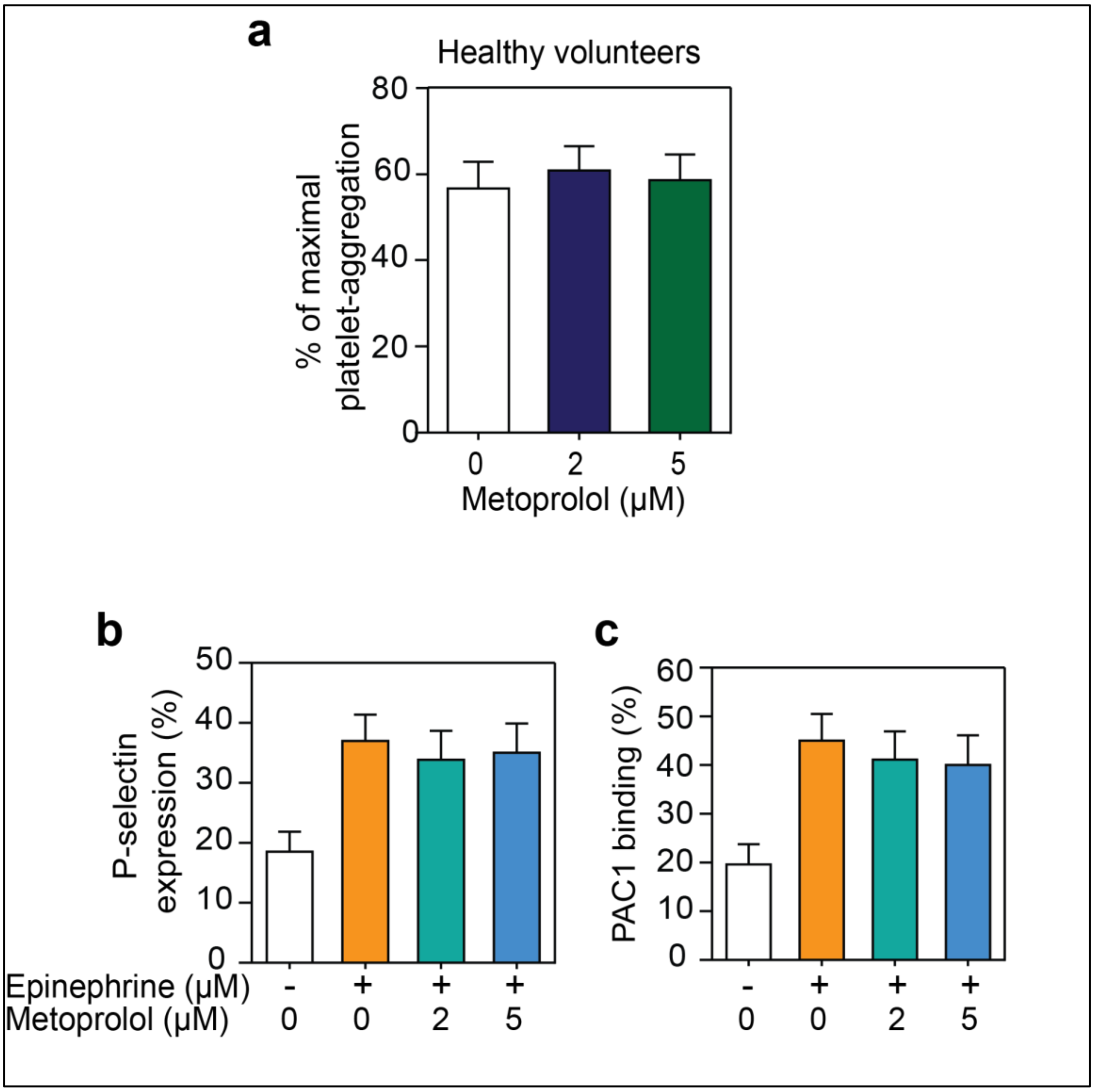

(**a**) Effect of metoprolol on maximal platelet aggregation on epinephrine-stimulated platelet rich plasma (PRP) from healthy volunteers (n=20). **(b-c)** Effect of metoprolol on platelet activation as determined by **(b)** surface expression of activated GP IIb/IIIa and **(c)** Surface expression of P-Selectin using flow cytometry from healthy donors (n=20). Data are means ± s.e.m.

Supplementary Figure 9. Metoprolol effect on mouse hemodynamics

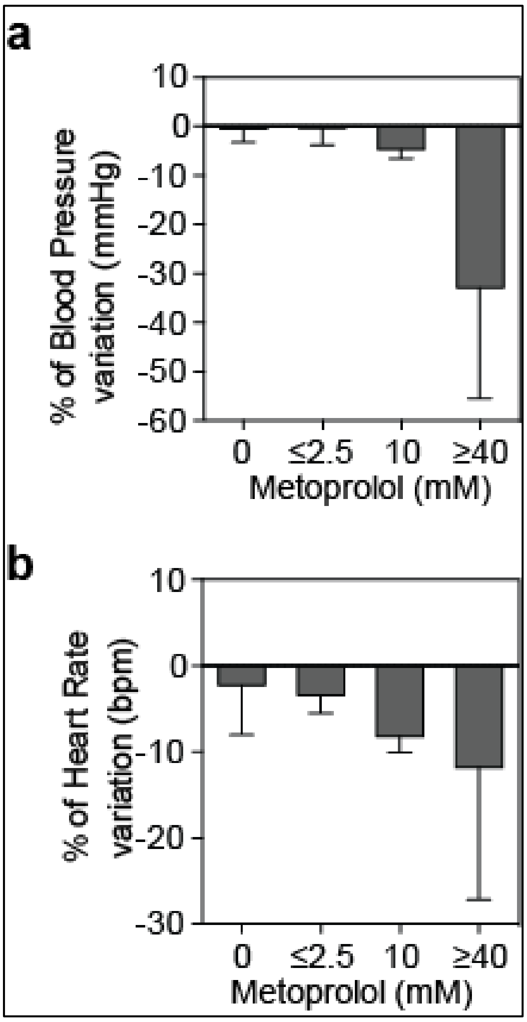

Hemodynamic effect of one single intravenous injection (50µL) through the femoral vein of metoprolol-tartrate at different concentrations. Blood pressure and heart rate were registered through the left arterial carotid artery with a PE-tubing catheter. (a) mean arterial pressure an mean arterial pressure and (b) heart rate as beats per minute (bpm). (n=3-4 individual animals per condition). Data are means ± s.e.m.

After this dose-response studies, the intravenous dose of metoprolol selected was 10mM. We identified this dose as the highest dose inducing a moderate effect on heart rate and blood pressure (i.e. <10% variation in both parameters from pre-dose).

Supplementary Table 1.

Population	Control<br>Median (IQR)	Metoprolol<br>Median (IQR)	p-value
**Leukocyte (x10^3)**	12.3 (10.3 – 14.7)	11.9 (9.3 – 13.7)	0.183
**Neutrophil (x10^3)**	9.5 (7.7 – 12.4)	9.1 (6.4- 11.3)	0.098
**Lymphocyte (Abs)**	1574 (1171 - 2509)	1754 (1265 - 2344)	0.164
**Monocyte (Abs)**	629 (482 - 837)	632 (504 - 851)	0.5
**Eosinophil (Abs)**	56 (27 - 162)	78 (35 - 168)	0.156
**Platelet (x10^5)**	224.5 (194.5 - 259)	218 (186 – 283.5)	0.753

Treatment comparison of leukocyte and subpopulations (neutrophil, lymphocyte, monocyte, eosinophil and platelet) count on admission in METOCARD-CNIC trial patients. Abs, stands for absolute count. IQR, stands for Interquartile range.
